# Business models for local energy market and circular economy establishment in municipalities: A case study in an Austrian municipality

**DOI:** 10.1016/j.heliyon.2023.e20776

**Published:** 2023-10-11

**Authors:** Matthias Maldet, Christoph Loschan, Daniel Schwabeneder, Georg Lettner, Hans Auer

**Affiliations:** Institute of Energy Systems and Electrical Drives, Energy Economics Group (EEG), Technical University of Vienna, Vienna, Austria

**Keywords:** Local energy markets, Circular economy, Municipal sustainable development, Portfolio and operation optimization, Energy policy implications

## Abstract

Energy communities often lack authority for establishment and operation management. Municipal authorities could take the role of such community operators. Therefore, Local Sustainable Municipalities are introduced, providing a local energy market on the municipal level with inclusion of sustainable resource utilization. The analyses include examinations of the scope of local markets in a municipality, portfolio investigations on different waste treatment plants and greywater system installation analyses. Furthermore, different adopted municipality strategies and their impact on municipal portfolio and market operation are examined. A clustering-based optimization framework for portfolio and market optimization is developed to perform these analyses. The proposed modeling approach leads to a significant model size reduction compared with hourly data optimization while providing location determination and portfolio estimation. The results indicate energy-sharing differences between municipal markets and local markets in energy communities. Decentralized energy provision is similar to centralized energy provision but on the municipal level. Furthermore, results show that waste incineration energy recovery can provide dispatchable low-emission energy with a high level of energy security and should be supported until the energy transition is more advanced. Finally, results on local strategies show that specific municipal goals always lead to increased costs for the municipality.

## Highlights

• The paper introduces Local Sustainable Municipalities (LSM).
• The methodology proposes a modeling framework for municipal technology.
• The paper assesses the impacts of various municipal sustainability strategies.
• LSM energy provision is similar to centralized provision on the municipal level.
• Municipal strategies lead to intended targets but increased costs.

## Graphical abstract

**Figure:**
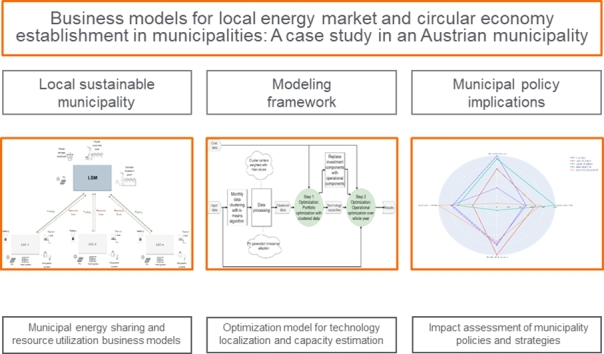

## 1 Introduction

Steadily increasing CO_2_ emissions [1] led to the European Commission (EC) introducing the Renewable Energy Directive [2]. This directive proposes cutting emissions by at least  by 2030. This should be achieved by developing technologies and incentives for renewable energy across multiple sectors across the European Union (EU). However, energy transition must go along with the United Nation Sustainable Development Goals (UN SDG) [3]. UN SDG goal 11 specifically addresses the urgency to form sustainable cities and communities. Thus the EU established the directives 2018/2001 [4] and 2019/944 [5] introducing legal frameworks for energy trading in energy communities. However, the scope of energy communities is broader than community formation on household level. Local municipal governments could establish decentralization in communities and emerge as operators for such communities. Therefore, Local Sustainable Municipalities (LSM) establishing a local market on the municipal level, are introduced in this work.

The local authorities in municipalities operate the LSM, wherein the market for electricity trading between participants is established. Thus, it is a superordinate market compared with local markets between consumers on the household level. LSM business models consider services apart from electricity trading. Resource management, like waste or sewage treatment, is often the area of responsibility of municipalities. However, resources are only treated without further advantage gain. Maldet et al. [6] proposed using recovered energy from resource treatment. LSM operations can have a large variety of options for energy recovery, with the main opportunity to sell recovered energy to LSM participants at reduced prices. The variety of options provide benefits for both municipalities and participants in gaining additional revenues for resource treatment and energy procurement at lower prices. Thus, LSM business models consider both energy operations and resource utilization. Therefore, this study analyzes the implementation of such business models into municipalities.

Introducing decentralized resource treatment facilities with implemented energy and resource recovery requires extensive infrastructure planning. Moreover, treatment and energy recovery strongly depend on local conditions such as the number of households, energy demand and potential technology installation at different sites, and locations within the municipality. Therefore, a portfolio analysis of the municipality, considering investment decisions in energy generation and conversion technologies, in treatment facilities is performed. The proposed analysis on LSM business models is applied to the municipality Breitenau am Steinfeld, Lower Austria [7].

The core objective of the analyses is to define the advantage and scope of the local market on the municipal level, which also considers resource utilization. Therefore, the impact of different resource treatment options and LSM objectives is addressed. Thus LSM business model investigation in a showcase municipality should encourage other municipalities to implement sustainable energy and resource business models. However, the goals to be considered in all analyses are the UN SDG. These core objectives lead to the following research questions.

• How can the introduction of an LSM contribute to the UN SDG?
• What is the impact of LSM business models on local energy and resource treatment portfolios?
• How do local markets over a whole municipality differ from local markets in (local) sustainable communities?
• How do different municipality strategies impact the portfolio and operations in the LSM?

## 2 State of research

### 2.1 Sustainable communities and municipalities

A large variety of research focuses on the establishment of sustainable communities and municipalities. According to Deakin [8], capacity planners are the fundamental agents to provide knowledge on urban generation processes. Salvia et al. [9] state that governments can be critical partners when moving toward resource efficiency in Europe. According to Battista et al. [10], local administration should have more responsibility in energy action plans. Trevisan et al. [11] emphasized the importance of local stakeholders' goals and their cooperation within a community. However, Åšlusarczyk and Grondys [12] proposed an essential role of municipalities belonging to economic zones, where UN SDG activities are performed. Moreover, Keskitalo and Liljenfeldt [13] found that the development of goals for sustainability leads to the requirement of priority setting. Thus sustainable processes in municipalities require detailed preliminary analysis. However, according to Bibri and Krogstie [14], multiple issues in future sustainable cities are uncertain, leading to the need for theoretically and practically convincing models. Krantz and Gustafsson [15] found challenges in UN SDG localization in sustainable operation integration and management of policies and ongoing efforts. Other work from Bibri and Krogstie [16] found a reorientation of sustainable cities to more sustainable strategies. Silva et al. [17] stated that major concerns in sustainable smart cities can include carbon footprint, waste management, sustainability and cost of reduction. Fenton et al. [18] introduced processes for energy and climate strategy development in Swedish municipalities with systematic stakeholder involvement, while Turvey [19] emphasized that understanding the relationship between sustainability and local development strategies is crucial for implementing such. De Vidovich et al. [20] identified key issues in developing valuable conditions for organizational and financial sustainability, whereas collaboration between different actors is crucial.

Research on sustainable municipalities and communities applies different strategies. According to Bibri [21], establishing a sustainable city has a high complexity level, leading to the requirement of interdisciplinary design strategies. Thornbush et al. [22] found that a combined mitigation-adaption method should be applied in sustainable city planning. Saadatian et al. [23] applied the sustainable city concept to university campuses by establishing sustainable community indicators. Petersen [24] introduced multiple layers for energy planning, with municipalities being a separate layer, while Dall'O et al. [25] proposed smartness indicators, with the main feature of flexibility. Neves et al. [26] applied a multi-criteria decision analysis to develop municipal energy action plans. Jeleński et al. [27] designed planning strategies by combining local air quality with global decarbonization goals, while Adil and Ko [28] focused on planning from the grid perspective. Moreover, Sperling et al. [29] reviewed eleven municipal energy plans, finding the need for better coordination in planning strategies. Wretling et al. [30] stated that the focus on municipal energy planning changed toward climate change mitigation. However, not all municipalities focus on this target. St. Denis and Parker [31] found a policy focusing on energy efficiency rather than expanding renewable energy. Brandoni and Polonara [32] found that the coordinator has a fundamental role in municipal policy setting. However, Johannsen et al. [33] stated that municipalities often lack the required planning tools for complex analyses. Moreover, Anastaselos et al. [34] found that local solutions are dependent on consumers' needs and priorities.

Many private projects and research projects already focus on community establishment. Österreichische Koordinationsstelle für Energiegemeinschaften [35] stated that energy trading projects by forming energy communities are increasingly established in Austria. Private incentives for sustainable communities such as GeWoZu [36] and Cambium [37] focus on a sustainable lifestyle rather than financial advantages. Research projects like Beyond [38] and Hybrid LSC [39] on further developing consumer-technology interaction in sustainable communities and municipalities.

### 2.2 Energy and resource utilization in municipalities

Energy and resource utilization research is often performed according to the UN SDG. Especially SDG 11 sustainable cities and communities [3], which directly refers to community operations. Fenton and Gustafsson [40] and Teixeira et al. [41] stated that municipalities and local actors could be vital for operationalizing the SDGs, while Gonzalez [42] highlighted municipal policy for sustainable growth. However, Scipioni et al. [43] emphasized the need to help municipalities in decisional processes. According to Han et al. [44], SDG policies must adapt to local conditions at the municipal level. Therefore, several research papers introduce different indicator systems. Almeida [45] identified social participation and governance as critical to achieving SDG 11. Moreover, Frare et al. [46] introduced six guiding axes: nature, public management, culture, sustainable education, accessibility and urban planning. Ilchenko and Lisogor [47] adopted hierarchical indexing considering economic, environmental and social components.

Energy and resource utilization in municipalities requires technology investment. Wang and Davies [48] highlighted green investment as an essential factor for sustainability in the supply chain. Sun et al. [49] examined the role of fiscal decentralization in promoting green investment, with local governments reinforcing the environmental rules for innovations. Moreover, Liu et al. [50] examined an emission reduction due to fiscal decentralization and renewable energy investment. Stakeholders must perform investments over multiple energy sectors. Alstone et al. [51] found a need for innovative approaches to electricity access in decarbonized energy systems. Yazdanie et al. [52] utilize local hydro, solar and waste resources in their study, leading to increased community self-sufficiency. Siraganyan et al. [53] proposed more robust policies to promote renewable energy systems.

Aside from energy investments, municipalities must apply resource treatment and management practices. Chen [54] stated that open information, integrated knowledge and responsibility are crucial for circular economy implementation. Reduction of waste and closing material cycles is vital, as stated by Mesjasz-Lech [55]. Thus, charging waste disposal costs should be done based on quantities, according to Alzamora and de V. Barros [56]. However, Periathamby [57] found that waste management structure is site-specific. Moya et al. [58] compared different waste-to-energy technologies finding high opportunities to obtain commodities such as materials and energy. Milutinović et al. [59] stated that anaerobic digestion is the best treatment practice from an environmental perspective. Moreover, Ohnishi et al. [60] highlighted the importance of waste treatment in the transition to low-carbon cities. Therefore, Alam and Qiao [61] analyzed waste treatment practices in a case study in Bangladesh, where they found high energy recovery potential from waste treatment. A similar study by Islam and Jashimuddin [62] found cost-effectivity in waste energy recovery. Suthar and Singh [63] examined further waste treatment energy recovery by compost biomass energy production. Furthermore, Zhang et al. [64] investigated the environmental benefits of sludge reuse. Wang and Davies [48] and Zhuang and Zhang [65] found that waste management and water management can potentially help in community operations. Xian et al. [66] stated that stricter regulations are mandatory to promote water-saving behavior in a sustainable society transition. Cureau and Ghisi [67] found water uses such as greywater to reduce potable water demand as an essential alternative. However, according to Piasecki [68], financial incentives are required to promote alternative sewage systems.

### 2.3 Community modeling

Research has widely adopted community optimization and business models. Reis et al. [69] and Capper et al. [70] examined energy communities' existing business and market models. Karami and Madlener [71] found increased household financial benefits by applying community business models. However, Maldet et al. [72] stated that business models depend on implemented legal frameworks on energy communities, which are adopted differently.

Moreover, various portfolio and investment decision optimization techniques have recently been developed. Casalicchio et al. [73] optimized operations and investments in communities. Xu et al. [74] developed a stochastic two-stage mixed-integer linear programming approach for multi-energy portfolio optimization while Kim et al. [75] performed a four-step process to consider uncertainties in decision-making. Meanwhile, Zhang et al. [76] and Zhou et al. [77] applied solving methods such as fuzzy programming and auto-regressive moving average in their optimization problems. Much research also focuses on the reduction of computational time in portfolio optimization problems. Sun et al. [78] stated that investment decisions considering every operating period are unrealistic. Liu et al. [79] and Härtel et al. [80] applied clustering to representative data for determining investment decisions. Palupi et al. [81] compared clustering algorithms to choose preferred market assets. Meanwhile, Pinel [82] proved that the K-means algorithm is the best clustering algorithm.

Gea-Bermudez et al. [83] stated that in energy communities, further research on multiple energy sector coupling is performed, as it increases renewable energy integration and demand flexibility. Multiple sector coupling frameworks in research already exist. Victoria et al. [84] provide a framework for network modeling of European electricity, heating, and transport sectors. Work from Rinaldi et al. [85] examined heat pump's flexibility to efficiently reduce total costs, while Lichtenwoehrer et al. [86] revealed district heating networks as feasible operations in multi-energy planning. Pavicevic et al. [87] found that multiple flexibility combinations provide the best short-term solutions in sector coupling. However, sector coupling should not only be limited to energy sectors. Fridgen et al. [88] proposed applying a holistic view on sector coupling along with information grids, while Maldet et al. [6] found that implementation of waste and water in sector coupling can increase efficiency.

### 2.4 Novelties and progress beyond the state of research

Various research already focuses on portfolio optimization and operational analysis of local markets in communities. However, adopting community business models with additional consideration of circular economy over a whole municipality needs further examination. The introduction of the LSM concept addresses these topics. Moreover, clustering-based energy system capacity localization methods and their particularities in resource treatment investment decisions in combination with operational optimization need further examination. Moreover, this research provides policy recommendations for municipalities adopting LSM business models.

The novelties and contributions beyond the state of the art of this work can be summarized as follows:

i) It introduces local markets on the municipal scope by introducing LSMs
ii) It investigates the impact of circular economy business models on municipal energy system operation
iii) It gives policy and strategy recommendations to municipalities adopting LSM business models
iv) It implements a clustering-based optimization framework for LSM capacity localization and operational analyses

## 3 Materials and method

The elaboration of the LSM research question requires the development of an optimization framework on sector coupling in municipalities with portfolio optimization functionalities. Therefore, the open-source framework resource utilization in sector coupling (RUTIS) [89] is extended to investment decisions. Maldet et al. [6] present the initial functionalities of the framework, which include sector coupling with resource utilization and energy community business models. Table 6 describes the nomenclature with the used mathematical symbols.

### 3.1 Optimization model framework

The model framework analyses consumer participation in local markets in the municipality. LSM participants are aggregated in communities, forming their own local market within the community. While these communities consider operation apart from energy trading, they are referred to as Local Sustainable Communities (LSC).

**Figure 1:**
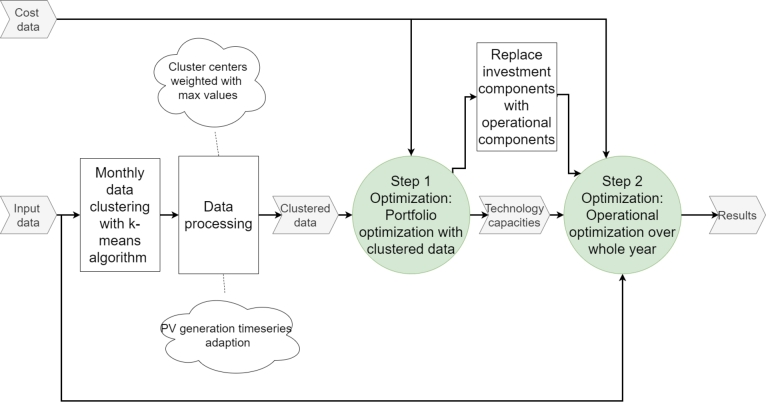
Optimization framework workflow.

Decentralized consumer community operations are performed in LSCs while multiple LSCs can interact in the LSM. Thus LSCs represent consumers in the LSM optimization model. Consumers are aggregated into four different LSCs with similar dimensions. Additionally, the fifth LSC with local municipality facilities is formed and represented in the model. The optimization model considers investment decisions on decentralized consumer technologies to cover the LSC energy and resource demands. Furthermore, the model includes investment decisions on the LSM level, which mainly include facilities for resource treatment. The primary goal of the investment decisions is determining the location of treatment facilities, while the exact capacity assessment has a subordinate role. The combination of multiple-sector investment decisions with community operations and business models results in a high optimization model complexity and long computing times. Therefore, an optimization framework approach decouples investment decisions and operational analyses. Fig. 1 presents the performed model workflow.

The portfolio optimization is performed with representative clusters of the data to reduce the model size of the optimization model. Annual input data must be preliminarily processed. Original input data in hourly resolution are transformed into 360 clusters, representing the whole year in a shorter period. Each cluster represents a representative time step of the original data. The clustering is performed with 30 clusters for each month to consider seasonal variance. The applied clustering method is a K-means algorithm, where cluster centers are used as adapted input data for the optimization model. However, more than clustering is needed to gain appropriate input data for the operational model. Cluster centers represent mean values, and required peak capacities for resource treatment are discarded in the clustering approach, resulting in non-feasible operational models due to insufficient treatment capacities. Therefore, the cluster centers are weighted with the maximum value of the input data. This approach provides model workflow improvements in terms of location determination but decreases accuracy in exact capacity determination. The clustered generation data are adapted to represent two-week generation input to limit the overestimation of PV generation.

The portfolio optimization is applied to clustered input data. Model results of the first step yield the required technology capacities with a major focus on localization. These capacities are provided as inputs to the operational model in step two together with original, hourly resolution input data.

In the second step, the optimization model is adapted so that no investment decisions must be performed. Thus the maximum technology operation capacities are set to the results of model step one. The second step investigates the detailed LSC operation on the LSM market over a year in hourly resolution. Operational optimization over longer periods is required due to seasonal variance and to assess resource planning over a longer period.

By performing the two-step optimization approach, capacity locations, capacity estimations and operations in the LSM can be assessed while keeping the model size acceptable. Detailed model methodology in the energy and resource sectors is presented in the following sections.

### 3.2 LSM model equations

**Figure 2:**
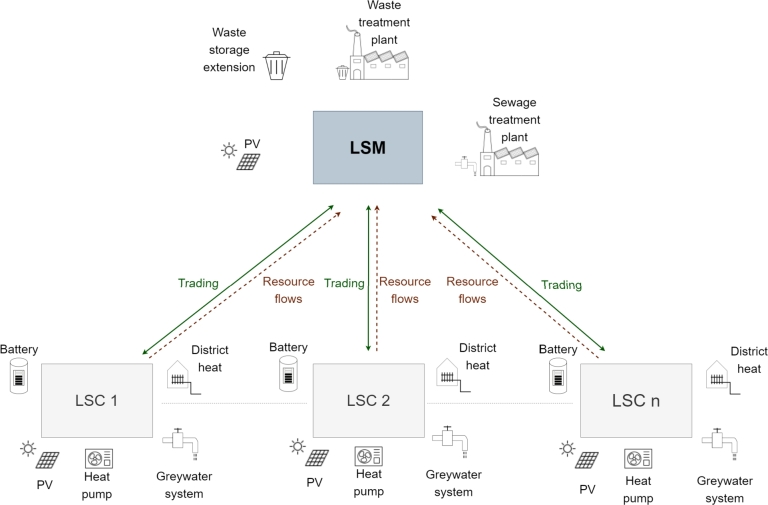
LSM operation.

LSM introduction and interaction with LSCs include multiple operations such as energy sharing, joint resource treatment and investment decisions. The functionalities presented in Fig. 2 must be considered in the investigation method.

LSM model analyses are performed as cost minimization problems with total costs composed of investment and operational costs, as presented in Equation (1). Investment costs are evaluated in the first modeling step, whereas operational costs are determined in the operation analysis.

(1) $$min(z)=min(c^{\mathrm{tot}})=min(c_{step1}^{\mathrm{inv}}+c_{step2}^{\mathrm{operational}})$$

The model considers technologies investment costs with annuities based on technology amortization rates $N_{l}$ and weighted average costs of capital WACC (assumed with ). The annuity factor for each technology $\alpha_{l}$ is calculated with Equation (2).

(2) $$\alpha_{l}=\frac{{\left(1+WACC\right)}^{N_{l}}\cdot WACC}{{\left(1+WACC\right)}^{N_{l}}-1}\;\forall l\in T$$

The model considers investment costs in the form of maximum capacity $x_{l}^{\max}$ based investment costs $C_{l}^{\mathrm{inv},\mathrm{var}}$ and fixed investment costs for installation or construction for each technology $C_{l}^{\mathrm{inv},\mathrm{fix}}$. As only a short period of the year is mapped in the first step of the optimization, the costs must be multiplied by a weighting factor $F^{\mathrm{year}}$ as the ratio of considered timesteps and total timesteps of a year. The relations are described in Equations (3) - (5).

(3) $$c_{l}^{\mathrm{inv},\mathrm{var}}=\alpha_{l}\cdot C_{l}^{\mathrm{inv},\mathrm{var}}\cdot F^{\mathrm{year}}\cdot x_{l}^{\max}\;\forall l\in T$$

(4) $$c_{l}^{\mathrm{inv},\mathrm{fix}}=\alpha_{l}\cdot C_{l}^{\mathrm{inv},\mathrm{fix}}\cdot F^{\mathrm{year}}\cdot bin_{l}^{\mathrm{install}}\;\forall l\in T$$

(5) $$F^{\mathrm{year}}=\frac{T^{\mathrm{cluster}}}{T^{\mathrm{year}}}$$

Equation (6) presents the total investment costs that can be calculated by a summation of investment costs of all technologies.

(6) $$c_{step1}^{\mathrm{inv}}=\sum_{l\in T}c_{l}^{\mathrm{inv},\mathrm{var}}+c_{l}^{\mathrm{inv},\mathrm{fix}}$$

Operational costs consist of operational technology costs (Equation (7)) and costs for external procurement (Equation (8)) of energy or resource at defined prices $\Pi_{k}^{\mathrm{procure}}$, such as electricity grid procurement or water pipeline procurement.

(7) $$c_{l}^{O\&M}=\sum_{t\in T}C_{l,t}^{O\&M}\cdot x_{l,t}\;\forall l\in T$$

(8) $$c_{k}^{\mathrm{procure}}=\sum_{t\in T}\Pi_{k}^{\mathrm{procure}}\cdot x_{k,t}\;\forall k\in E$$

Moreover, revenues can be generated by the sale of energy or resources, as presented in Equation (9).

(9) $$rev_{f}^{\mathrm{feedin}}=\sum_{t\in T}\Pi_{f}^{\mathrm{feedin}}\cdot x_{k,t}\;\forall k\in E$$

The model considers environmental aspects of the LSM in the form of CO_2_ emissions. They are calculated by summation of technology and external source procurement emissions by calculation with CO_2_ factors $F^{\mathrm{CO2}}$ (Equation (10)) and monetarized by CO_2_ prices (Equation (11)). The emission costs are counted as operational costs. For the construction of capacities, no emissions are assumed, as the optimization focuses on environmental performance in the operation rather than in the whole life cycle.

(10) $$em_{t}^{\mathrm{CO2}}=\sum_{l\in T}F_{l}^{\mathrm{CO2}}\cdot x_{l,t}+\sum_{k\in E}F_{k}^{\mathrm{CO2}}\cdot x_{k,t}$$

(11) $$c_{t}^{\mathrm{CO2}}=em_{t}^{\mathrm{CO2}}\cdot \Pi^{\mathrm{CO2}}$$

Equation (12) presents the summation of all kinds of operational costs to total operational costs.

(12) $$c_{step2}^{\mathrm{operational}}=\sum_{l\in T}c_{l}^{O\&M}+\sum_{k\in E}c_{k}^{\mathrm{procure}}-\sum_{f\in F}rev_{f}^{\mathrm{feedin}}+\sum_{t\in T}c_{t}^{\mathrm{CO2}}$$

Detailed information on the considered costs differs between sectors. Furthermore, model constraints are defined to describe the LSM system. Both are presented in the following section.

### 3.3 LSM sectoral costs and constraints

Model constraints consider technology limitations, conversion relations and storage equations as presented in [6]. Moreover, equilibrium constraints for each sector must be considered, which is dependent on the available technologies in the respective sector. For balance rules, it must further differ between LSC balance rule and LSM balance rule. Balance rules are graphically represented in the Appendix. Furthermore, energy recovery equations are presented in the Appendix. The goal is always to cover pre-defined demands. Therefore, sets representing the LSCs and LSM are defined, with the LSM represented at each LSC position.

#### 3.3.1 Waste sector

The LSM is responsible for waste management. Accruing waste from each LSC is allocated to the LSM at a particular geographical position. Moreover, sludge from sewage treatment is counted as waste and must be treated. An investment decision in waste treatment technologies is performed in each LSM position. Waste can be incinerated to recover electricity and heat, whereas  of energy recovery utilization is assumed. The use of recovered energy depends on aspects such as grid infrastructure and contracts with plant operators. Therefore, sensitivity analyses on recovered energy utilization are conducted to assess the impact of different shares of recovered energy utilization. Recovered energy is allocated to the corresponding sectors. However, emissions occur due to the share of non-biodegradable waste. Therefore, emissions of  of CO_2_ emissions per kg waste are assumed independent of the considered waste treatment option. These emissions are assumed based on IEA Bioenergy [90] and the share of biowaste and sludge in the municipality. A further option for waste treatment is anaerobic digestion. Generated gas can be incinerated in gas CHP plants or sold on the gas wholesale market. For gas CHP, an investment decision must be performed. Further options are limited external disposal and reduction of waste. Reduction is monetarized by the value of Cialani and Mortazavi [91]. As some positions must have waste treatment facilities, waste transport between LSM positions is enabled. By binary variables, simultaneous transport input and output is disabled to prevent circular waste flows. This results in the following balance rule for waste in Equation (13)

(13) $$\begin{array}{cc} D_{LSC_{m},t}^{\mathrm{wasteIn}}+m_{m,t}^{\mathrm{sludge}} & +\sum_{n\in M,n\neq m}m_{m,n,t}^{\mathrm{wastetrans},\mathrm{in}}\\ & =m_{m,comb,t}^{\mathrm{waste}}+m_{m,AD,t}^{\mathrm{waste}}+m_{m,disp,t}\\ & +m_{m,red,t}+\sum_{n\in M,n\neq m}m_{m,n,t}^{\mathrm{wastetrans},\mathrm{out}} \end{array}$$

#### 3.3.2 Water and sewage sector

Similar to waste, sewage from the LSCs is aligned to the LSM at each position. An investment decision in sewage treatment plants is determined to assess capacity and position of the facility. Like for waste treatment, sewage treatment investment by municipalities is financial participation to larger treatment plants. Electricity is required as an additional input for sewage treatment and must be provided by the LSM. Outputs from sewage treatment include recovered water (assumption  use of recovered water) and sludge (as input to the waste sector). Recovered water can be sold to LSCs at defined prices $\Pi_{i,m}^{waterrecovery}$ depending on the distance between the sewage treatment plant and LSC.

A certain amount of the water demand can be covered by greywater system installation. The share of greywater in sewage is assumed with  whereas kitchen sewage is not considered greywater because of the additional required efforts to extract food remainings [92], [93]. Moreover, it is assumed that only  of the total water demand can be covered by greywater [94]. However, the model must perform an investment decision on greywater installation, with potential system installation in each household. This results in a mixed integer problem, considering the installed greywater systems as integer variables $int_{i}^{\mathrm{greywater}}$. The integer variable is limited by the number of households $N_{i}^{\mathrm{household}}$ in the LSM as presented in Equation (14). The total greywater that can be used is calculated by Equation (15), considering the unit volume of .

(14) $$int_{i}^{\mathrm{greywater}}\leq N_{i}^{\mathrm{household}}$$

(15) $$v_{i,greywater,t}\leq int_{i}^{\mathrm{greywater}}\cdot V^{\mathrm{greywater},\mathrm{unit}}$$

The remaining water must be covered by pipeline purchase, resulting into the following water balance rule in Equation (16).

(16) $$D_{i,water,t}=v_{i,waterpipe,t}+v_{i,greywater,t}+\sum_{m\in M}v_{i,m,sewage,t}^{\mathrm{water}}$$

Sewage can be transmitted to other positions to prevent multiple treatment plant installations. This leads to the sewage balance rule in Equation (17).

(17) $$v_{m,sewage,t}-v_{m,sewagetreat,t}+\sum_{n\in M,n\neq m}v_{m,n,sewage,t}^{\mathrm{trans},\mathrm{in}}=\sum_{n\in M,n\neq m}v_{m,n,sewage,t}^{\mathrm{trans},\mathrm{out}}$$

#### 3.3.3 Electricity sector

The electricity sector for LSCs considers investment in PV and battery capacities. Excess generation can be fed into the electricity grid. Moreover, electricity can be sold at predefined prices to the LSM, like presented in Equation (18).

(18) $$rev_{i,t}^{\mathrm{elec2LSM}}=q_{i,LSM_{i},t}^{\mathrm{elec2LSM}}\cdot \Pi_{i}^{\mathrm{elec2LSM}}\;\forall i\in C$$

LSCs can procure electricity at defined prices and efficiencies from the LSM. Prices and efficiencies are dependent on grid length between the geographical LSC position in the municipality, represented with purchase of LSC at position *i* from LSM at position *m*. This is presented in Equation (19).

(19) $$c_{i,m,t}^{\mathrm{LSM2elec}}=\frac{q_{i,m,t}^{\mathrm{LSM2elec}}}{\eta_{i,m}^{\mathrm{LSM2elec}}}\cdot \Pi_{i,m}^{\mathrm{LSM2elec}}\;\forall i\in C,m\in M$$

Procurement prices are lowered by reduced grid tariffs based on the grid section, resulting in position-dependent electricity grid procurement electricity prices.

The LSCs must provide electricity to heat pumps for heat generation if these are installed. The remaining electricity is procured from the electricity grid, whereas additional emissions for grid procurement are assumed as  according to [95]. This results in the LSC balance rule in Equation (20).

(20) $$\begin{array}{cc} q_{i,PV,t}+q_{i,elgrid,t}^{\mathrm{procure}}+q_{i,bat,t}^{\mathrm{out}} & +\sum_{m\in M}q_{i,m,t}^{\mathrm{LSM2elec}}\cdot \eta_{i,m}^{\mathrm{LSM2elec}}\\ & =D_{i,elec,t}+q_{i,HP,t}^{\mathrm{elec}}+q_{i,elgrid,t}^{\mathrm{feedin}}+q_{i,LSM_{i},t}^{\mathrm{elec2LSM}} \end{array}$$

The LSM can sell electricity to LSCs. This electricity can come from LSC sales to the LSM but also from energy recovery of waste incineration. Moreover, the LSM must provide the required electricity for sewage treatment. However, this only accounts if an investment decision is performed in the LSM at a certain position. This results in the balance rule in Equation (21).

(21) $$\begin{array}{cc} q_{m,PV,t}+q_{m,elgrid,t}^{\mathrm{procure}}+q_{LSC_{m},t}^{\mathrm{elec2LSM}} & +q_{m,comb,t}^{\mathrm{elec}}\\ & +q_{m,CHP,t}^{\mathrm{elec}}=\sum_{i\in C}\frac{q_{i,m,t}^{\mathrm{LSM2elec}}}{\eta_{i,m}^{\mathrm{LSM2elec}}}+q_{m,sewage,t}^{\mathrm{elec}} \end{array}$$

#### 3.3.4 Heat sector

The internal option for the LSCs to cover the heating demand is the installation of heat pumps. However, as heat can be recovered from waste treatment, the analyses must consider district heating installations. The model applies investment decisions in district heating systems. With district heating capacity installed, LSCs can procure recovered heat at pre-defined LSM prices $\Pi^{\mathrm{LSM2heat}}$ at distance-dependent efficiencies $\eta_{i,m}^{\mathrm{LSM2heat}}$. The procured heat is limited by the installed capacity, as presented in Equation (22).

(22) $$q_{i,m,t}^{\mathrm{LSM2heat}}\leq q_{i,DH}^{\mathrm{installed}}$$

This leads to the LSC heat balance rule in Equation (23).

(23) $$D_{i,t}^{\mathrm{heat}}=\sum_{m\in M}q_{i,m,t}^{\mathrm{LSM2heat}}\cdot \eta_{i,m,t}^{\mathrm{LSM2heat}}+q_{i,HP,t}^{\mathrm{heat}}$$

LSM heat can be generated by energy recovery with waste incineration or biogas CHP. Non-usable heat is considered exhaust heat, resulting in Equation (24).

(24) $$q_{m,comb,t}^{\mathrm{heat}}+q_{m,CHP,t}^{\mathrm{heat}}=\sum_{i\in C}\frac{q_{i,m,t}^{\mathrm{LSM2heat}}}{\eta_{i,m,t}^{\mathrm{LSM2heat}}}+q_{m,exhaustheat,t}$$

### 3.4 Case study

The LSM business models' application and optimization framework are tested in Breitenau am Steinfeld in Lower Austria [7]. The municipality consists of 1581 residents living in 730 households. Moreover, the case study considers public buildings. Table 1 presents the aggregation of residents and public buildings. The aggregation is conducted by forming five LSCs within the municipality. More detailed information on scenario settings is presented in the Appendix.

**Table 1:** Municipality configuration.

LSC	Residents	Households
1	372	163
2	453	230
3	417	200
4	339	137
5 (public)	-	-

#### 3.4.1 Scenario settings

For the elaboration of the research questions, the study establishes four different scenario settings in the municipality. The “Trading” scenario setting considers sensitivity analyses on PV capacity in the LSCs, focusing on the impact on LSCs without their own PV installation possibility. Thus the effect of trading over the local LSM market is analyzed. Scenario setting “Circular economy” examines waste treatment portfolio optimization by considering waste incineration and anaerobic digestion as significant options. In the “Greywater” scenario setting, investment decisions in separate greywater systems and impact on costs and water household are assessed. Finally, scenario setting “Policy and strategy” considers different municipality strategies by monetarizing targets. Table 2 summarizes the scenario settings.

**Table 2:** Scenario settings.

Scenario setting	Description
SC1: Trading	Impact assessment of trading on local markets
SC2: Circular economy	Waste treatment portfolio optimization
SC3: Greywater	Profitability analysis on separate greywater systems
SC4: Policy and strategy	Analysis on different policies and strategies by monetarizing objectives
Self-sufficiency policy	Strategy by modeling high electricity grid procurement costs
Low-emission policy	Modeling of high CO_2_ prices to lower emissions
Local-efficiency policy	Keeping energy and resources in the municipality loop by penalizing electricity grid feedin and exhaust heat
2040 scenario	Scenario setting to give policy recommendations: CO_2_ neutral electricity mix and CO_2_ prices of

Moreover, key performance indicators (KPI) are defined to compare community operations in different scenario settings. These KPIs are summarized in Table 3. The non-local LSM utilization parameter is defined in Equation (25), considering the ratio of fed-in electricity $q_{elgrid}^{\mathrm{feedin}}$, exhaust heat $q_{exhaustheat}$, disposed waste $m_{disp}$ multiplied with its heating value $H_{S}^{\mathrm{waste}}$ and externally sold gas $q_{gassale}$ to sum of total electricity $d_{el,total}$ and heat demand $D_{heat}$, accruing waste $M_{waste}^{\mathrm{accruing}}$ and total generated gas $q_{wasteAD}^{\mathrm{gas}}$. The total electricity demand is set together of pre-defined electricity demand $D_{el}$, electricity demand for heat pumps $q_{HP}^{\mathrm{elec}}$ and electricity demand for sewage treatment $q_{sewage}^{\mathrm{elec}}$ according to Equation (26).

(25) $$\begin{array}{c} \sigma^{nonLocal}=\frac{q_{elgrid}^{\mathrm{feedin}}+q_{exhaustheat}+H_{S}^{\mathrm{waste}}\cdot m_{disp}+q_{gassale}}{d_{el,total}+D_{heat}+H_{S}^{\mathrm{waste}}\cdot D_{waste}^{\mathrm{accruing}}+q_{wasteAD}^{\mathrm{gas}}} \end{array}$$

(26) $$\begin{array}{c} d_{el,total}=D_{el}+q_{HP}^{\mathrm{elec}}+q_{sewage}^{\mathrm{elec}} \end{array}$$

**Table 3:** Key performance indicators.

KPI	Unit	Description
Investment costs	€	Total costs for technology investment
Operational costs	€	Total costs in the LSM operation
Total costs	€	Total costs (= sum of investment costs and operational costs)
Total CO_2_ emissions	t	Total emissions from all LSC and LSM processes
Electricity grid purchase		Total procurement from electricity grid
Electricity grid feeding		Total electricity fed into the grid
Exhaust heat		Non-usable heat from energy recovery
Non-local LSM utilization of energy and resources	-	Non-LSM use parameter according to Euqation (25)
Electricity to LSM		Total electricity sold from LSCs to LSM market
LSM to electricity		Total electricity procured by LSCs from LSM market

#### 3.4.2 Model validation

The validation compares the developed optimization framework to the same scenario setting with a four-hour mean value optimization for the model validation. In the four-hour mean value scenario, investment decisions and operational analysis were evaluated in one optimization step. Both optimization practices lead to similar technology installation and to the same localization. Thus the model is applicable to the intended analyses. The Appendix presents a detailed methodology for the model validation.

## 4 Results

This section summarizes the results of the scenario settings presented in Table 2. Each scenario setting is represented by a chapter in this section, beginning with the trading analyses in Section 4.1, followed by the circular economy results in Section 4.2 and results on greywater utilization in Section 4.3. Section 4.4 concludes the result section.

### 4.1 LSM technology and market implementation

The results in this section present the impact of PV installation in the LSM with and without the establishment of electricity trading on LSM market. Resource utilization is implemented as joint treatment, but recovered energy procurement is exempted. Moreover, the results of a sensitivity analysis in the same setup with no PV installation by LSC1 and LSC4 are presented.

PV investment is executed to the maximum possible capacities at all LSM positions, independent of trading implementation. Introduction of trading has only a minor impact on the total costs, leading to a cost reduction of  from  to . Emissions decrease by  to . Electricity grid consumption decreases by  to . Electricity sale from consumers to the LSM market is conducted at  per year, while electricity procurement is only performed at  per year. The remaining energy not procured by participants is used by the LSM to operate the sewage treatment plant.

**Figure 3:**
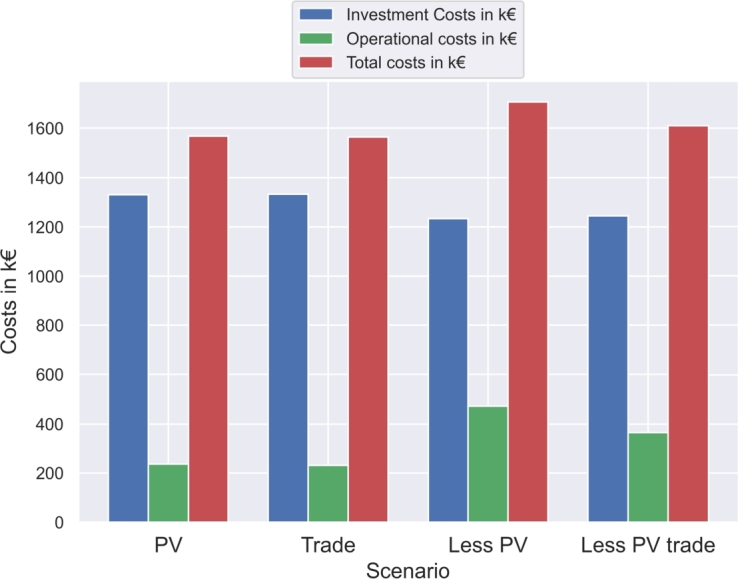
Comparison of total costs for the LSM setup (PV), trading implementation (Trade) and the sensitivity analysis without trading (Less PV) and with trading (Less PV trade).

The impact changes in the sensitivity analysis with less PV. Without trading, the total costs would increase by  respectively . Electricity grid consumption rises by  () and the total emissions increase by  (). Introduction of trading over the LSM market leads to a cost decrease of  (), an emission reduction of  () and a grid consumption decrease of  (). Fig. 3 presents the total costs for different PV and trading settings, showing a shift from operational costs to investment costs with PV installation and a cost increase without LSM market trading.

Compared with the setting with the maximum possible PV installation, the sale of electricity via the LSM market increases by a factor 34 to  while LSM electricity procurement increases by a factor 112 to .

**Figure 4:**
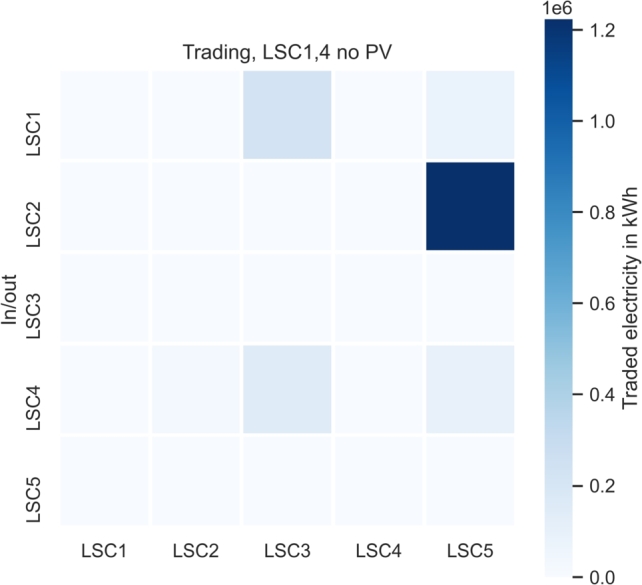
LSM trading flow allocation for sensitivity analysis.

Fig. 4 presents the sensitivity analysis trading allocation, where LSCs without their own PV installation benefit from additional local market procurement due to increased purchases from other LSCs. However, the figure shows that LSCs without PV are not the highest profiteer from trading.

### 4.2 Circular economy in LSM

The setup in the previous section is extended to waste and sewage treatment energy and water recovery implementation. The analyses focus on treatment plant localization and treatment portfolio determination. For the comparison between different portfolios, the studies consider a sensitivity analysis on energy recovery from waste incineration. Moreover, the alternative treatment option of waste anaerobic digestion is investigated as an additional setting adaption.

Energy recovery in the LSM has an impact on treatment facility localization. Without energy recovery, the installation of treatment plants is conducted at LSC3, as it is the position with the shortest transportation distance to other locations. Sewage treatment plant localization has no impact on the model outcome, whereas with enabled trading, sewage and waste treatment plants are installed simulataneously. Moreover, trading changes the treatment plant position from LSC3 to LSC4, as LSC4 has the lowest PV installation potential compared to the electricity demand.

Different waste incineration energy recovery utilization yields technology installation differences. Table 4 presents the results of the performed sensitivity analysis.

**Table 4:** Waste incineration energy recovery sensitivity analysis.

Setting	Trade, no recovery	20% recovery	50% recovery	90% recovery
Battery in	146	119	60	39
District heat in	0	47	114	205
LSM electricity sale in	21.2	1.3	0.4	0
Total emissions in t	650	600	532	447
Total costs in M€	1.56	1.47	1.42	1.37

Battery installation decreases, replacing the time-flexibility of battery storages by waste storages. District heat installation increases as more capacity is needed to procure higher amounts of recovered heat from treatment processes. Moreover, electricity sale by LSCs via the LSM market is minimized. Emissions and total costs decrease with increasingly recovered electricity and heat, leading to cost efficiency with recovered energy from waste treatment.

**Figure 5:**
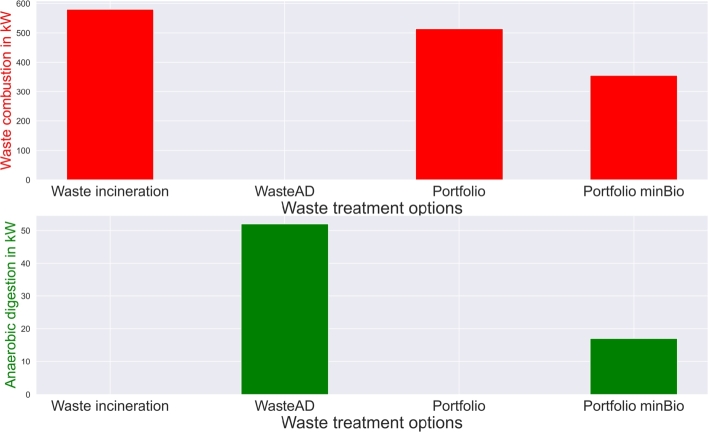
Circular economy waste treatment installation at different available waste treatment options and goals.

**Figure 6:**
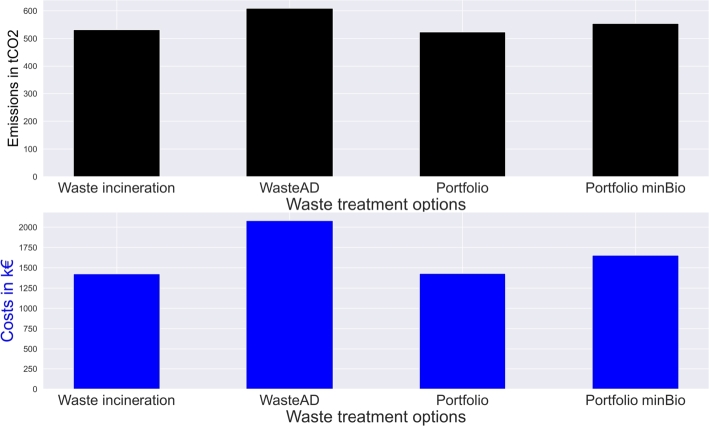
Circular economy costs and emissions at different available waste treatment options and goals.

Replacement of waste incineration by anaerobic digestion (“WasteAD”), and joint consideration of both treatment options (“Portfolio”) have a direct impact on total costs and emissions. Figure 5, Figure 6 show the comparison between the technology installations, emissions and costs of different waste treatment options; costs and emissions directly correlate in the setups.

Primarily waste anaerobic digestion leads to a cost increase of  and an emission increase of . Portfolio analyses with competing technologies lead to waste incineration plant installations only. High gas prices on its wholesale market do not lead to additional investment in anaerobic digestion plants. By setting biowaste treatment targets for biogas production (“Portfolio minBio”),  of the waste treatment facility capacities are anaerobic digestion plants.  are waste incineration capacities, while the remaining  of waste are reduced which leads to an efficient means of a circular economy. However, such goals increase the costs by  and the emissions by  due to increased electricity grid consumption. Exhaust heat and electricity grid feedin are reduced with increased use of anaerobic digestion.

### 4.3 Greywater utilization

This section presents the analyses on separate greywater systems. The waste portfolio optimization setup from Section 4.2 is extended to greywater installation options. Analyses with and without minimum greywater utilization goals are performed. Such goals are necessary for greywater systems to be installed due to the high investment costs of separate systems and the efficient usage of water recovered from sewage treatment. However, without sewage treatment water recovery, greywater installation is done at 99 households in the LSM. The same results emerge for minimum greywater utilization goals. Water procurement from alternative options to pipeline purchase increases from  to . Required sewage treatment capacity decreases by  to  compared with scenarios without greywater utilization. Moreover, waste incineration capacity decreases by  to  due to less sludge emergence. This leads to a decrease of district heat installation by  () due to less recovered heat. Table 5 compares the results from SC3 (greywater utilization) to settings from SC1 (trading without energy recovery) and SC2 (waste portfolio optimization).

**Table 5:** Setup comparison between casual LSM operation without energy recovery, waste portfolio analysis with energy recovery and greywater utilization.

Setting	Trade, no recovery	Waste portfolio	Greywater utilization
Elec. grid purchase in	2114	1609	1642
Elec. grid feedin in	7806	8167	8142
Exhaust heat in	0	500	461
Total emissions in t	650	523	514
Total costs in M€	1.56	1.43	1.46

**Table 6:** Model parameters and decision variables.

**Sets**		
C	LSCs	index: i
J	Sectors	index: j
M	LSM positions	index: m
E	External sources	index: k
T	Available technologies	index: l
*T*	Total timesteps	index: t

**Parameters**		
*WACC*	Weighted average cost of capital	%
*N*	Amortization period	-
*C*^inv,var^	Capacity-based share of investment costs	€ per [*technology*]
*C*^inv,fix^	Fixed share of investment costs	€
*C*^o&M^	Operational costs	€ per [*technology*]
*N*^household^	Number of households per LSC	-
*D*_*j*_	Demand or accruing resource per sector	[*sector*]
*V*^greywater,unit^	Maximum volume of one greywater system	
*F*^CO2^	Emission factor	t per [*technology*]
*T*^cluster^	Number of time steps in clustering step	-
*T*^year^	Number of time steps in operational step	-
*F*^year^	Factor for shorter optimization period	-
Π^procure^	Price for external procurement	€ per [*source*]
Π^feedin^	External feedin tariff	€ per [*source*]
Π^elec2LSM^	Electricity LSM sale tariff	€ per
Π^LSM2elec^	Price LSM electricity procurement	€ per
Π^CO2^	CO_2_ price	€per t
$H_{S}^{\mathrm{waste}}$	Heating value waste	
$M_{waste,max}^{\mathrm{trans}}$	Transport capacity waste	


**Variables**		
*c*^tot^	Total costs	€
$c_{step1}^{\mathrm{inv}}$	Investment costs step 1	€
$c_{step2}^{\mathrm{operational}}$	Operational costs step 2	€
*α*	Annuity factor	-
*x*^max^	Technology capacity investment	[*technology*]
*bin*^install^	Binary variable technology investment	-
*x*_*l*_	Technology flow	[*technology*]
*x*_*k*_	External source flow	[*source*]
*c*^inv,var^	Variable investment costs	€
*c*^inv,fix^	Fixed investment costs	€
*c*^O&M^	Operational costs	€
*c*^procure^	External procurement costs	€
*rev*^feedin^	External feedin revenues	€
*em*^CO2^	CO_2_ emissions	t
*c*^*CO*2^	Emission costs	€
*m*^sludge^	Sludge to waste	
*m*^wastetrans,in^	Waste transported input	
*m*^wastetrans,out^	Waste transported output	
$m_{comb}^{\mathrm{waste}}$	Incinerated waste	
$m_{AD}^{\mathrm{waste}}$	Digested waste	
$q_{AD}^{\mathrm{gas}}$	Generated biogas	
*m*_*disp*_	Externally disposed waste	
*m*_*red*_	Reduced waste	
*int*^greywater^	Installed greywater systems	-
*v*_*greywater*_	Volume greywater systems	
*v*_*waterpipe*_	Pipeline procured water	
*v*_*sewage*_	Accruing sewage	
*v*_*sewagetreat*_	Treated sewage	
$v_{sewage}^{\mathrm{water}}$	Recovered water	
$v_{sewage}^{\mathrm{trans},\mathrm{in}}$	Transmitted sewage input	
$v_{sewage}^{\mathrm{trans},\mathrm{out}}$	Transmitted sewage output	
*q*^elec2LSM^	Electricity sold to LSM	
*rev*^elec2LSM^	Revenues electricity to LSM	€
*q*^LSM2elec^	Electricity procured from LSM	
*c*^LSM2elec^	Costs electricity procurement from LSM	€
*η*^LSM2elec^	Efficiency LSM electricity procurement	-
*q*_*PV*_	PV generation input	
$q_{elgrid}^{\mathrm{procure}}$	Electricity grid procurement	
$q_{bat}^{\mathrm{in}}$	Battery input	
$q_{bat}^{\mathrm{out}}$	Battery output	
$q_{HP}^{\mathrm{elec}}$	Electricity heat pump	
$q_{HP}^{\mathrm{heat}}$	Heat generation heat pump	
$q_{elgrid}^{\mathrm{feedin}}$	Electricity grid procurement	
$q_{comb}^{\mathrm{elec}}$	Waste combustion electricity	
$\eta_{comb}^{\mathrm{elec}}$	Electric efficiency waste combustion	-
$q_{comb}^{\mathrm{heat}}$	Waste combustion heat	
$\eta_{comb}^{\mathrm{heat}}$	Thermal efficiency waste combustion	-
*em*_*comb*_	Emissions waste combustion	t
$share_{comb}^{\mathrm{usable}}$	Usable energy recovery combustion	-
*share*^biodegradable^	Share biodegradable waste	-
*bin*^wastetrans,out^	Binary transport variable	[0,1]
$q_{CHP}^{\mathrm{elec}}$	Gas CHP electricity	
$q_{CHP}^{\mathrm{heat}}$	Gas CHP heat	
$q_{sewage}^{\mathrm{elec}}$	Electricity demand sewage treatment	
*q*^LSM2heat^	LSM heat procurement	
*η*^LSM2elec^	Efficiency LSM heat procurement	-
*q*_*exhaustheat*_	Exhaust heat	
*q*_*gassale*_	Sold gas at market	
*d*_*el*,*total*_	Total variable electricity demand	
*σ*^*nonLocal*^	Non local energy and resource use	-

Electricity grid consumption increases by  and total costs increase by  compared with the same setting without greywater goals. However, fed-in electricity (), exhaust heat () and total emissions () decrease. Compared to settings without energy or water recovery, greywater utilization leads to an improvement in all presented KPIs.

### 4.4 LSM policy and strategy

In the final result section, different LSM policies and strategies are compared. The setup considers all waste portfolio options. Section 4.4.1 presents the impact of different policies and strategies on the KPIs and investments while Section 4.4.2 investigates other impacts in terms of total costs, emissions, self-sufficiency and local energy and resource utilization.

#### 4.4.1 Policy impact

**Figure 7:**
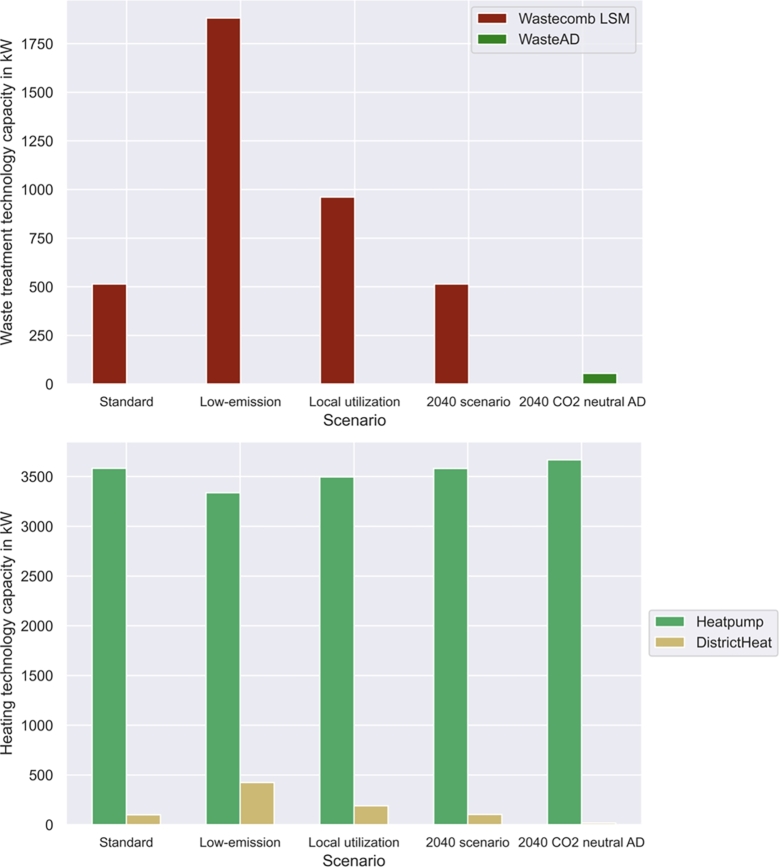
Strategy and policy impact on waste and investments.

**Figure 8:**
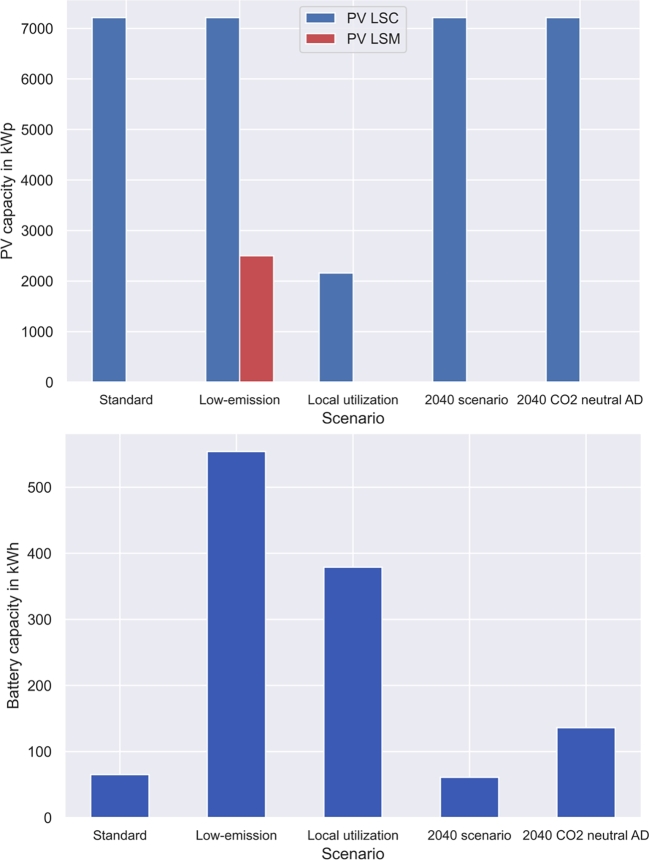
Strategy and policy impact on electricity investments.

This section presents the impact of different LSM policies presented in Table 2. Self-sufficiency and low-emission policies lead to similar results, as the electricity grid is the strongest source of emissions in the LSM. Thus, for policy comparisons, low-emission scenarios are considered. The 2040 policy investigations consider analyses on the same settings as in basic policies, with only differences of zero-emission electricity mixes and high CO_2_ prices. Furthermore, a scenario with a zero-emission waste anaerobic digestion and recycling combination is considered in a different strategy. The impacts on investments are presented in Figure 7, Figure 8.

The installed waste treatment plant is strongly dependent on the LSM policies and strategies. Without specific targets, waste incineration is the installed waste treatment technology. Low-emission strategies lead to increased incineration plant installations at various LSC positions to avoid transport of waste. However, this leads to increased overall waste incineration capacities of . Local utilization also leads to additional waste incineration capacities of  to treat waste when the recovered energy is needed in the LSM. Anaerobic digestion is only utilized in 2040 scenarios if it is possible to make the process emission-free through additional recycling.

PV installation decreases from  by  to  in the local utilization scenario to avoid electricity feedin as much as possible. Moreover, the LSM installs additional PV capacity in the low-emission scenario to prevent electricity grid consumption. Other scenarios consider the maximum possible PV installation at LSCs of  without separate LSM PV generation. Battery investments increase by  in low-emission policies and by  in local-utilization policies. These two strategies thus lead to the highest increases in battery investments. Strategies with zero-emission anaerobic digestion lead to additional battery investment due to lower waste treatment process efficiency. Heat pumps are the main source of heat in all scenarios. However, installed district heat capacities vary depending on the employed strategy. Additional waste incineration plant installation leads to higher energy recovery and thus to increased district heat installation.

**Figure 9:**
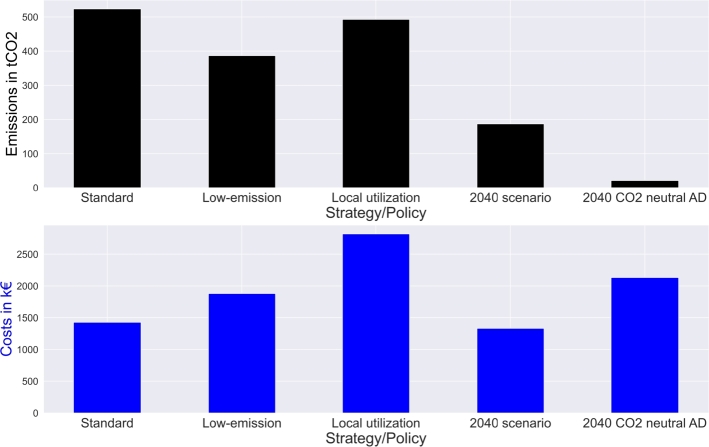
Strategy and policy impact on costs and emissions.

Fig. 9 presents the impact of employed strategies on total costs and emissions.

Low-emission policies lead to an emission decrease of  and to additional costs of  owing to more required investment. Local utilization strategies lead to sharply rising costs by  but only slightly declining CO_2_ emissions by . The 2040 scenarios lead to cost decreases () due to assumed lower electricity prices and emission decreases () due to zero-emission electricity mixes. Low-emission strategies in 2040 are not impacted due to low emissions in centralized generation technologies. However, a reduction in emissions of  compared to standard policies can be achieved by establishing zero-emission anaerobic digestion technologies. The disadvantage is an accompanying cost increase of  compared to traditional policies.

#### 4.4.2 Policy comparison

**Figure 10:**
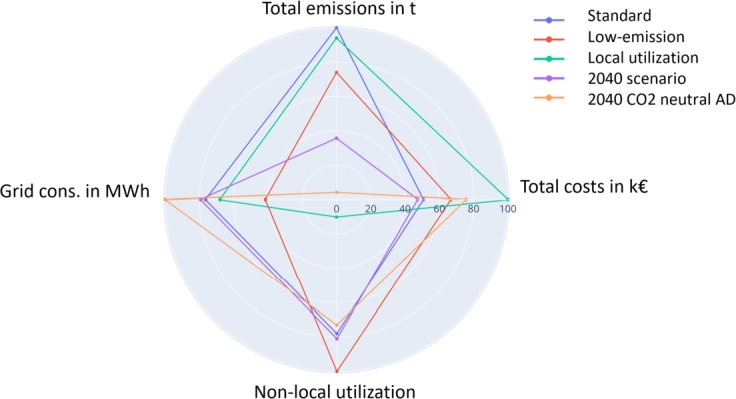
Policy impact comparison on self-sufficiency and local utilization as percentage of maximum achievement.

Finally, results in this section present a comparison of the proposed LSM policies and strategies. Fig. 10 shows the comparison based on total costs, total emissions, electricity grid consumption and non-local utilization based on the percentage of impact compared to maximum impact. Grid consumption represents the self-sufficiency of the LSM.

Only price drops can achieve cost reduction in centralized generation, with the 2040 scenario being the only setup leading to lower costs. All policies can achieve emission reduction, although the costs required for reduction differ. Grid consumption is the lowest for low-emission scenarios, as the electricity grid is the main source of emissions (except from 2040 scenarios). The results show a similar local resource utilization for all strategies except for those designed to reach this goal. Particular strategies lead to the highest local utilization, with a parameter improvement of . However, costs and emissions increase disproportionately to achieve the goal.

## 5 Discussion

This section discusses the synthesis of the significant results in Section 4, beginning with a discussion on market scopes of the introduced LSM market in Section 5.1. Building upon that, Section 5.2 discusses the impact of implemented energy and circular economy business models regarding the UN SDG [3]. Finally, section 5.3 provides a discussion of different LSM goals and strategies.

### 5.1 Local market scopes

LSM markets emerge as superordinate markets to local markets in communities, such as LSCs. Results in Section 4.1 show a significant operation on the LSM market. The provided platform for local trading can emerge as a cost-reduction opportunity for participants by providing options to sell their excess energy and procure energy from LSM technologies or other participants. Furthermore, with LSM introduction, the municipal government could emerge as a driver for community establishment. Results in Section 4.1 did not consider the utilization of resource treatment energy recovery, leading to a high share of electricity sale of participants via the LSM market. Thus, the LSM marketplace has similar functionality as a local market in communities, with the difference being that the LSM has a high electricity demand for processes such as sewage treatment. Excess electricity can thus be used in a community where most participants have a surplus of PV electricity simultaneously.

Moreover, Fig. 4 shows that the LSM market could be an opportunity for consumers without PV installation options, as they can benefit from the procurement of cheaper energy. Community building of people of the same income classes and thus social exclusion can be prevented by a public marketplace. Even though directives of the EU on energy communities [4], [5] demand non-discriminatory access of consumers to local energy communities, consumers might be deterred from insisting on such laws.

The impact of electricity sale on the LSM market changes when energy recovery utilization is implemented. Table 4 shows a decline in electricity sold by LSM participants with an increasing share of used energy recovery from resource treatment. Therefore, if a municipality can utilize high-capacity energy generation technologies, the local market changes from providing an option for electricity sale to provide cheap energy for participants. This leads to an inversion of the energy flows in the LSM. In such cases, the LSM is more of a centralized energy provision approach implemented in decentralized local markets. Thus, the LSM market has a different scope than local markets on the community level leading to a distinct division of competencies. Local markets in communities provide more of a socially encouraged energy sharing for lower energy amounts, whereas LSM markets encourage decentralization and energy recovery of resource treatment facilities. However, both markets implemented simultaneously can provide many options for consumers to take part in local energy markets.

### 5.2 Sustainable LSM energy and resource utilization

LSM utilization became an opportunity for consumer engagement in sustainable operations. Sharing of renewable energy in the LSM can contribute to sustainable operations, as presented by the results in Section 4.1. Results in Section 4.2 show further opportunities for energy recovery utilization. However, the impact is dependent on the utilized share of energy recovery (Table 4) and the provided waste treatment options (Figure 5, Figure 6). In a fully considered waste treatment portfolio, waste incineration is the technology to be considered due to its higher efficiency compared to waste anaerobic digestion. The environmental performance of both technologies is the same, as the non-biodegradable share of waste leads to emissions in both technologies due to alternative disposal in anaerobic digestion. Without trading and energy recovery, the installation of treatment plants is undertaken at the site with the shortest distance to other consumers. Trading implementation changes the site to the location with the highest excess energy. Energy availability has a higher impact than transport distances. However, it is unrealistic for each municipality to invest in its treatment facility. Therefore, the investment should be seen as municipalities' financial participation in a multi-municipality treatment plant. Moreover, the required grid infrastructure for recovered electricity and heat distribution must be installed. Thus, despite high waste treatment decentralization potential, implementation barriers in supplementary infrastructure availability and high investment costs might emerge.

Greywater utilization is not performed from a financial perspective. However, as water efficiency is increased, greywater can emerge as an opportunity to prevent water scarcity. Therefore, greywater installation could gain significance from an environmental perspective. Furthermore, technology installation is dependent on resource utilization. Table 4 shows decreasing battery installation with rising energy recovery share. Waste storages replace battery storages for time-flexibility in the energy system. Moreover, district heat installation is crucial to use recovered heat from waste incineration.

From the perspective of the UN SDG [3], LSM introduction is a direct contribution to SDG 11, sustainable cities and communities. Moreover, contributions to SDG 7, affordable and clean energy, emerge by providing clean energy over a local market. SDG 6, clean water and sanitation, and SDG 12, responsible consumption and production, are addressed by the implementation of the circular economy business model implementation. All LSM actions can contribute to SDG 13, climate action, on the municipal level.

### 5.3 Impact of LSM goals and policies

Results in Section 4.4 provide insight into LSM policy impacts. Fig. 7 shows inefficient waste incineration facility installation to reach specific municipality goals by better-timed operation. Moreover, Fig. 9 indicates that LSM strategy employment always leads to cost increases. However, emissions can be reduced by appropriate strategies. Fig. 10 shows that low-emission policies lead to high self-sufficiency in current setups owing to emission-intensive electricity mixes. However, to reach emission reduction, the accruing cost increase must be covered. The difference must either be covered by municipalities or by governmental actions in the form of higher CO_2_ prices. Furthermore, Fig. 10 shows that inefficient local utilization strategies lead to high cost and emission increases. Therefore, such strategies should not be pursued with it.

Future policies lead to emission decreases due to zero-emission electricity grid procurement, as presented in Fig. 9. However, the 2040 emission reduction policies only have a minor impact on the municipal level. Process efficiency improvement, such as waste anaerobic digestion with alternative treatment of non-biodegradable waste can provide opportunities for further emission decrease. However, technology improvement leads to rising costs, which municipalities must cover. In summary, municipalities must have clear objectives. Moreover, municipalities must expect increasing costs to be covered when implementing strategies to achieve specific objectives.

Employed strategies can further be affected by the European legal framework in the Renewable Energy Directive (RED) [2]. According to the directive, energy from waste incineration should only count as renewable by preliminary removing of fossil share of materials [96], as waste is never incinerated without such share [97]. However, waste treatment plant operators have concerns over energy security [98]. The LSM analyses indicate significant contributions of waste incineration to total emissions. These emissions are low compared to emissions from electricity grid procurement, leading to higher total emissions in the short term by prohibiting waste incineration. Moreover, in contrast to natural gas, waste is a safely available resource for energy generation. However, future decarbonized energy systems should consider alternative zero-emission waste treatment options, immediate omission of waste incineration plants could backfire in terms of emission reduction and energy security. Therefore, until a phase-out of fossil fuels in the electricity mix can be achieved, waste incineration could emerge as a bridge technology.

## 6 Conclusions

This work introduces local energy markets with resource utilization on the municipal level by adopting the concept of an LSM. LSM business models are applied in the municipality Breitenau am Steinfeld, forming an LSM out of residents, aggregated to five LSCs. As there are no specific local limitations or constraints in the municipality, the modeling approach can also be applied to municipalities with similar scope.

LSM introduction can be crucial to contribute to the UN SDG. Moreover, the LSM could provide a marketplace for the sale of municipality residents' excess energy and for the procurement of recovered energy from waste treatment. However, the local LSM market is similar to a centralized approach, implemented in decentralized energy systems due to significant energy generation capacities from waste treatment. Energy trading decreases from  per year to almost zero trading if energy recovery is fully utilized. Therefore, the focus of trading should be set on LSC operations with smaller scopes of communities. The LSM market should be set up as a higher-level local market with the provision of decentralized energy and resource treatment to LSM members at predefined prices.

Planning of waste and sewage treatment facilities should be performed based on energy availability rather than transport distances. Resource treatment can be efficiently implemented in LSM business models, with waste incineration being the most efficient treatment option. The implementation can lead to cost reductions of about . Moreover, it can provide a dispatchable energy generation option of safely available resources and should thus not be prohibited until the energy transition is further advanced. Anaerobic digestion can emerge as a treatment option in low-emission scenarios when emissions from non-biodegradable waste can be avoided in the process. However, the utilization of energy recovery requires efficient decentralization of treatment facilities and the availability of grid infrastructure. Greywater system installation is uneconomical but could become an option in addressing water scarcity issues.

LSM low-emission policies can contribute to emission reductions up to  but they lead to increased costs up to . Local resource utilization policies are cost-efficient and environmentally inefficient and should thus be avoided. However, municipalities must set clear energy and resource utilization goals when adopting LSM business models and bear costs to reach municipal environmental goals.

The developed clustering-based optimization framework is efficient in model size reduction, leading to model-solving times under one hour. The proposed method can appropriately determine the technology and treatment facility location. However, the required data processing for treatment facility capacity determination decreases accuracy in energy generation and storage portfolio analysis.

Limitations emerge in the complexity of the considered sectors and technologies in the analyses. Thus, hydrogen technologies are not yet considered in the approach. Moreover, a significant limitation of the approach is in the scope of the LSM. Usually, municipalities would rather contribute to regional plants than invest in their waste and sewage treatment plants. In the proposed LSM business models, the municipalities only invest in a share of the plants. However, detailed analyses should consider a joint investment with other municipalities. Furthermore, LSM operation should continue beyond municipality boundaries.

Therefore, future research should consider the interaction between multiple municipalities. Moreover, more detailed analyses of municipal waste treatment facilities should be conducted, which emphasizes on waste. Additionally, the extension of the analyses to hydrogen technologies should be further examined. Finally, life cycle assessment investigations could be performed within the investment decisions in the municipality.

**Nomenclature**

## CRediT authorship contribution statement

**Matthias Maldet:** Conceptualization, Data curation, Formal analysis, Investigation, Methodology, Software, Validation, Visualization, Writing – original draft, Writing – review & editing. **Christoph Loschan:** Data curation, Investigation, Methodology, Resources, Validation. **Daniel Schwabeneder:** Data curation, Investigation, Methodology, Resources, Validation. **Georg Lettner:** Conceptualization, Formal analysis, Funding acquisition, Project administration. **Hans Auer:** Conceptualization, Formal analysis, Supervision.

## Declaration of Competing Interest

The authors declare that they have no known competing financial interests or personal relationships that could have appeared to influence the work reported in this paper.

## Figures and Tables

**Figure A.11:**
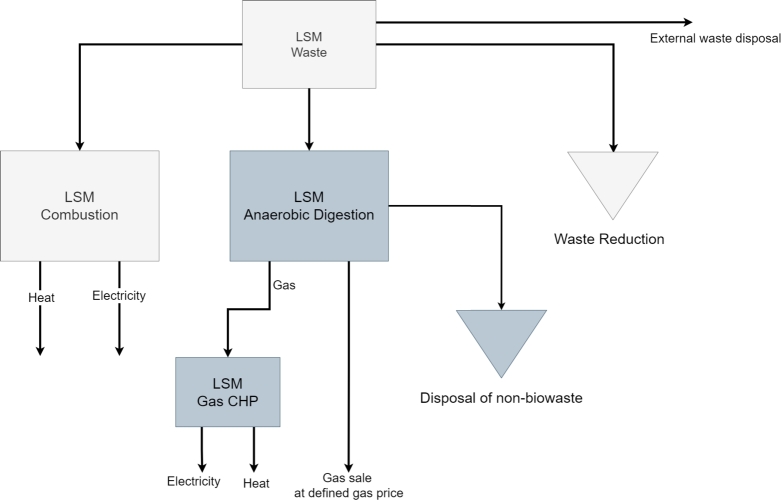
Waste treatment options.

**Figure A.12:**
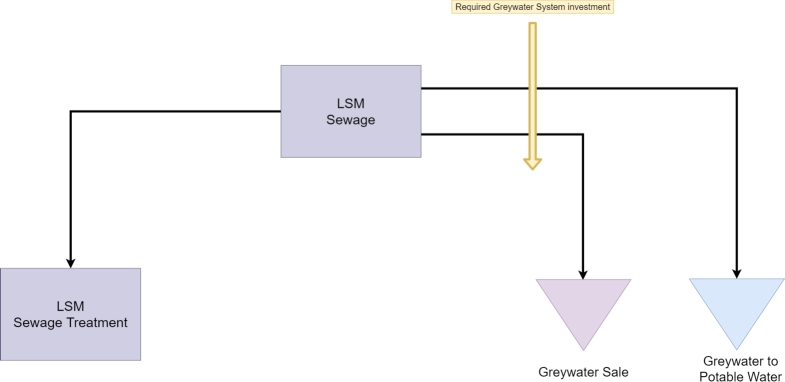
Sewage treatment options.

**Figure A.13:**
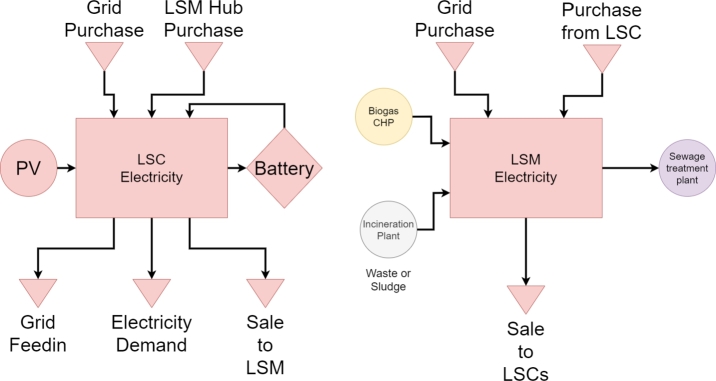
Electricity inputs and outputs.

**Figure A.14:**
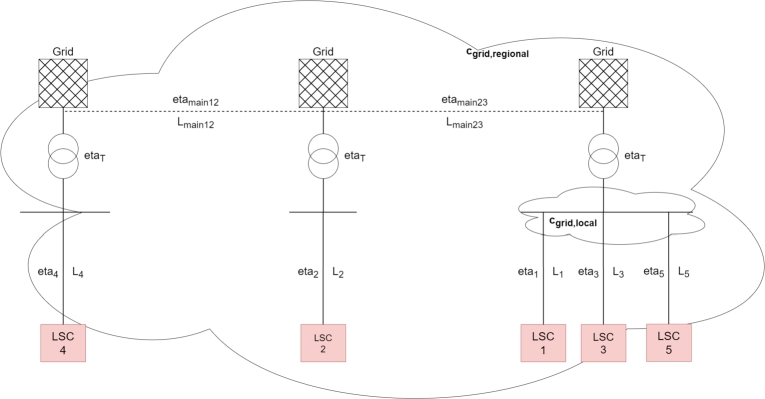
LSM electricity grid.

**Figure A.15:**
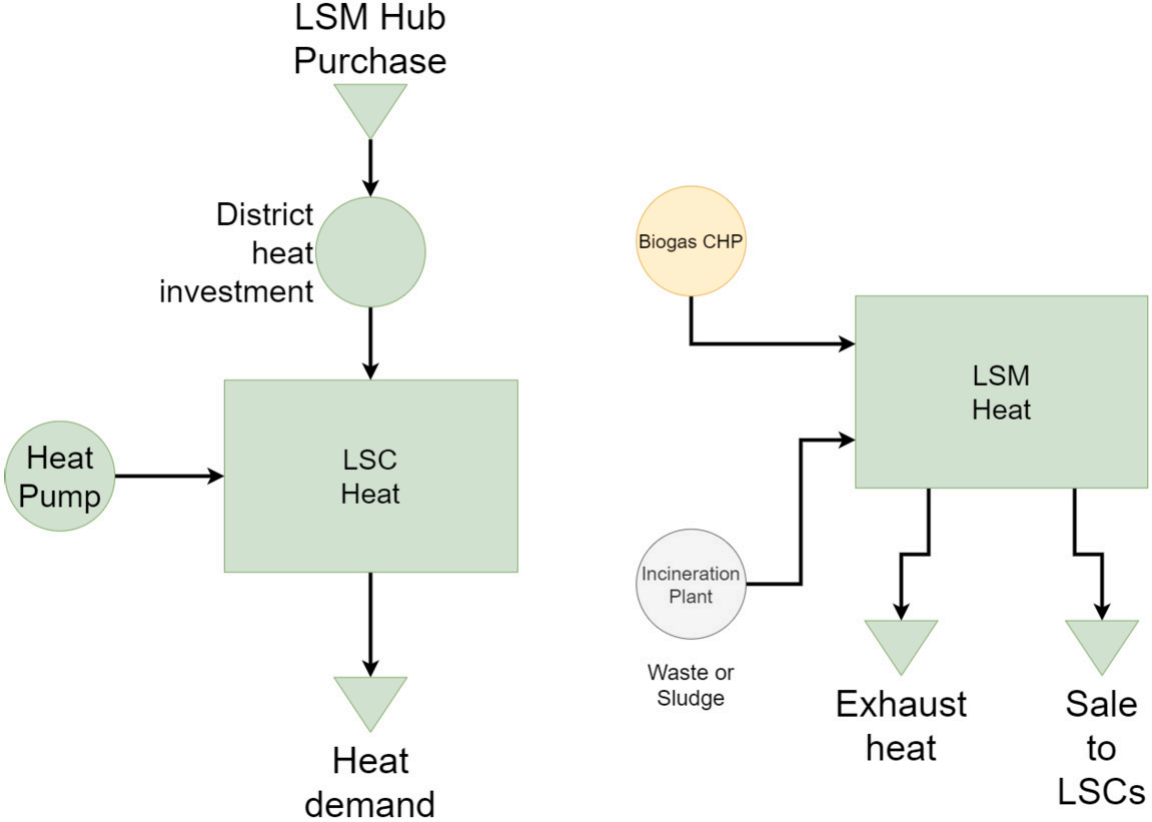
Heat inputs and outputs.

**Table B.7:** Municipality configuration.

LSC	Electricity in	Heat in	Water in	Waste in t
1	359	2200	17112	167
2	441	2680	20838	204
3	402	2467	19182	188
4	328	2005	15594	153
5	166	585	1669	136

**Table B.8:** Municipality technology assumptions.

LSC	PV in	Battery in	Heat pump in	District heat in
1	1630	1630	1141	1141
2	2162	2300	1610	1610
3	2000	2000	1400	1400
4	1010	1370	1370	1370
5	412	190	400	400

**Table B.9:** Tariff assumptions.

Tariff	Value
Electricity purchase	
Electricity purchase 2040 [107]	
Electricity feedin	
Electricity LSM sale	
Electricity LSM purchase local	
Electricity LSM purchase regional	
LSM heat purchase	
Water pipeline purchase	
Water LSM purchase	
Water LSM purchase at same position	
Waste transport costs	
Waste disposal costs	
CO_2_ price	
Gas price low	
Gas price high	

**Table B.10:** Investment cost assumptions.

Technology	Fixed costs	Variable costs	Amortization period in years	Source
PV			20	[109]
Battery			10	[110]
Heat pump			8	[111], [112]
District heat connection			20	[113]
Waste combustion			10	[114]
Waste storage			10	[115], [116]
Sewage treatment plant			20	[117], [118]
Anaerobic digestion			10	[119]
Gas CHP	-		16	[120]
Greywater system		-	15	[121]

**Table C.11:** Validation results: four hour resolution approach.

Tech.	LSC1	LSC2	LSC3	LSC4	LSC5	Sum
PV in	1630	2162	2000	1010	412	7214
Battery in	21	22	8	8	0	59
District heat in	0	126	105	0	0	231
Heat pump	804	853	796	732	214	3399
Waste comb in	0	0	580	0	0	580
Sewage treat in	0	0	9	0	0	9

Total costs in €	1,201,762					

Computation time	10 h 7 min 5 sec ()					

**Table C.12:** Validation results: clustering-based decoupling approach.

Tech.	LSC1	LSC2	LSC3	LSC4	LSC5	Sum
PV in	1630	2162	2000	1010	412	7214
Battery in	0	16	9	10	3	38
District heat in	0	119	112	0	0	231
Heat pump	816	875	803	743	217	3454
Waste comb in	0	0	580	0	0	580
Sewage treat in	0	0	9	0	0	9

Total costs in €	1,358,035					

Computation time	0 h 49 min 36 sec ()					

## Data Availability

Data included in article/supplementary material/referenced in article.

## Appendix A Balance rule representation

In this section, the balance rules described in Section 3 are graphically presented. Furthermore, additional model equations required for understanding the technical functionalities are emphasized.

### A.1 Waste sector

Waste is set together of municipal waste and sewage sludge. Emerging waste is assumed as time-constant input time series. Basic assumptions consider the incineration of both resources. Electricity and heat can be recovered. However, it can be assumed that not all energy can be reused in the municipality. This results in the following waste incineration equations.

(A.1) $$q_{comb,t}^{\mathrm{elec}}=\eta_{comb}^{\mathrm{elec}}\cdot m_{comb,t}^{\mathrm{waste}}\cdot share_{comb}^{\mathrm{usable}}$$

(A.2) $$q_{comb,t}^{\mathrm{heat}}=\eta_{comb}^{\mathrm{heat}}\cdot m_{comb,t}^{\mathrm{waste}}\cdot share_{comb}^{\mathrm{usable}}$$

(A.3) $$em_{comb,t}=F_{comb}^{\mathrm{CO2}}\cdot m_{comb,t}^{\mathrm{waste}}\cdot share_{comb}^{\mathrm{nonbiogene}}$$

Extensions of waste portfolios consider anaerobic digestion for biogas generation, as presented in Fig. A.11.

Generated biogas presented in Equation (A.4) can be incinerated or sold externally.

(A.4) $$q_{wasteAD,t}^{\mathrm{gas}}=m_{wasteAD,t}^{\mathrm{waste}}\cdot \eta_{wasteAD}\cdot H_{S}^{\mathrm{waste}}\cdot share^{\mathrm{biodegradable}}$$

Incinerated biogas is determined by investment decisions in gas CHP plants. Relations are similar to Equations (A.1) and (A.2), with the significant difference that for biogas incineration, no direct emissions are assumed. However, only biodegradable waste can be digested. The share of non-biodegradable waste must be thermally treated, resulting in the same emissions as waste incineration. Apart from extensions to zero-emission anaerobic digestion with combined recycling of non-biodegradable waste, the same emissions are assumed for both treatment options.

Waste can be transported between different LSM positions by waste trucks at defined distance-dependent costs and emissions to prevent treatment at multiple sites. However, to avoid circular flows of waste, input and output for waste transport in the same time steps are prohibited by the introduction of binary-blocking constraints.

(A.5) $$m_{m,n,t}^{\mathrm{wastetrans},\mathrm{out}}\leq bin_{m,n,t}^{\mathrm{wastetrans},\mathrm{out}}\cdot M_{trans}^{\mathrm{waste},\max}\;\forall m,n\in M,m\neq n$$

(A.6) $$m_{m,n,t}^{\mathrm{wastetrans},\mathrm{in}}\leq (1-bin_{m,n,t}^{\mathrm{wastetrans},\mathrm{out}})\cdot M_{trans}^{\mathrm{waste},\max}\;\forall m,n\in M,m\neq n$$

### A.2 Water and sewage sector

Water and sewage are implemented using a circular economy approach. Sewage from LSCs is jointly treated by the LSM, whereas recovered water can be sold by the LSM to the LSCs at distance dependent prices. Sludge as a by-product is assigned to the waste sector. Similar to waste, sewage can be transmitted between different positions to prevent multiple sewage treatment plant installations. The major difference is that for sewage transmission, no costs and emissions are assumed due to the pipeline-based transmission system. Moreover, the implementation of greywater systems is investigated as presented in Section 3.3.2. The setup for the water and sewage circular economy implementation is presented in Fig. A.12.

### A.3 Electricity sector

Fig. A.13 presents electricity inputs and outputs of the LSCs and LSM.

The goal is to cover the given electricity demand for all consumers. Installed PV systems and market procurement from the LSM can provide LSC electricity. The remaining electricity can be procured from the public electricity grid. Excess electricity can be sold on the LSM market or fed back into the electricity grid at pre-defined prices. Moreover, battery investment is included in the analyses. LSM electricity can be procured over the LSM market and from the electricity grid. Recovered electricity from waste or biogas incineration is assigned to the LSM. The LSM must provide the required electricity for sewage treatment. The remaining electricity can be sold to the LSCs at distance-dependent efficiencies and grid prices. To understand the setting of efficiencies and prices, Fig. A.14 presents the electricity grid setup of the LSM.

Transformer efficiencies and efficiencies depending on the line length are assumed. Grid tariffs are set based on grid level, differing between local and regional grid tariffs.

### A.4 Heat sector

Fig. A.15 presents the balance rules in the heat sector.

Installed heat pumps can generate LSC heat to cover the pre-defined heat demand. Moreover, investment decisions in district heat connections are performed. Heat can be procured from local LSM markets if investment in district heat connection is performed. The source of the procured heat can be either waste or biogas incineration, as it can be seen in the balance rule of the LSM. Procurement costs are dependent on grid length, similar to the electricity sector, without the assumption on the difference in distance-dependent grid tariffs for heat.

## Appendix B Consumer, cost and technology assumptions

The different assumptions considered for the input parameters of the consumers are presented in this section. The municipality provided data for the public facilities and resident statistics. Demand and technology assumptions are taken based on household statistics.

For the demand profiles, the aggregations of residents in LCSs presented in Table 1 are considered. The percentual share of household sizes is assumed according to [99]. Electricity consumption per consumer is considered based on [42], whereas heat consumption relies on the assumptions of [100]. Annual water consumption is assumed based on [101], while accruing waste input comes from statistics of the considered municipality. This leads to the demand assumptions in Table B.7.

Technology data are assumed based on the household size. PV generation profiles are considered based on [102] while COP profiles of heat pumps are taken based on [103]. The maximum technology capacity assumptions for each LSC are summarized in Table B.8.

Existing waste storage capacities are assumed with 1/12 of the accruing waste, with potential expansion of . Maximum resource treatment and gas CHP capacities for investment decisions are assumed to be sufficiently high to guarantee model feasibility. The same applies to the input parameter of maximum waste transport mass. Waste transport distance varies between  and  based on geographical distances in the municipality, with LSC3 having the shortest distance to other LSCs in sum. Transport emissions are directly related to transport distances. For electricity lines,  losses per  are assumed, according to [104]. Additionally, transformer losses of  are taken based on [105]. For heat procurement, losses of  per km are assumed, according to [106].

Tariffs for external procurement and LSM market operation are summarized in Table B.9.

Technology operational costs are considered to have the same values as presented in [6]. Finally, Table B.10 presents the taken investment costs. Amortization periods are assumed based on [108].

## Appendix C Model validation

The model validation was performed by comparing the developed clustering-based optimization framework to the same scenario setting with a four-hour mean value optimization approach. Consequently, a whole year is optimized without the decoupling of portfolio optimization and operational analysis. However, both methods are simplifications compared to hourly resolution optimization annually. The model comparison is tested for a basic LSM scenario where technology investment and trading are enabled. Furthermore,  of recovered energy from waste treatment can be utilized. In the model validation investigations, waste incineration is the only available treatment option in the portfolio. Table C.11, Table C.12 show the portfolio and total cost results. Moreover, the computational times of both optimization runs are presented.

PV is installed at the maximum possible value in both cases. Thus, the PV capacity is determined by the physical building constraints, rather than the modeling approach. However, for the proposed investigation setup, the method is appropriate for PV capacity assessment. Battery investment was underestimated in the optimization frameworks' method, resulting in  less investment. Moreover, the allocation of batteries to LSCs differs in the approaches. District heat was estimated to have the exact capacity in both optimization practices, with the emergence of a minor difference in capacity allocation. However, district heat was installed at LSC2 and LSC3 in both cases. Heat pump capacity was set  higher in the proposed optimization framework, but the ratios of installation at LSCs were similar. Waste and sewage treatment plants were determined at the same capacity and at the same location in both approaches, leading to the optimization framework method being an appropriate simplification for treatment facility determination.

The total cost difference in both approaches was  respectively . This was due to differences in the operation in both simplified methods. However, a considerable advantage emerged in the model size reduction. While the four-hour mean value approach leads to computation times over  the proposed optimization framework practice could be performed in less than . This corresponds to a computational time reduction of .

Overall, the clustering-based optimization framework technique was efficient in terms of technology localization. The total cost deviation was slight compared to the advantage of the model size reduction. The core objectives of the analyses are more dependent on technology and treatment facility allocation than on the exact determination of total costs. Moreover, both methods are a simplification and thus it must be assumed that neither can lead to  exactly incurred costs. Analyses also consider more complex circular economy investigations such as anaerobic digestion and greywater utilization. In such scenario settings, model size reduction gains even higher significance. Thus the developed method is applicable for the elaboration of the research questions.
